# Determinants of improving the relationship between corporate culture and work performance: Illusion or reality of serial mediation of leadership and work engagement in a crisis period?

**DOI:** 10.3389/fpsyg.2023.1135199

**Published:** 2023-03-15

**Authors:** Jakub Michulek, Lubica Gajanova, Anna Krizanova, Margareta Nadanyiova

**Affiliations:** Department of Economics, Faculty of Operation and Economics of Transport and Communications, University of Zilina, Zilina, Slovakia

**Keywords:** company culture, work performance, leadership, work engagement, mediation

## Abstract

The disease COVID-19 has had an impact on the lives of all people in the world. It also had a great impact on the world economies themselves. There are several scientific publications on the impact of the COVID-19 disease on the work performance of employees, while researchers have focused on less traditional factors such as corporate culture, leadership, or work engagement as well. Corporate culture influences the shared values or behavior of employees at the workplace and creates an environment in which employees work. Through leadership, managers should be able to motivate their employees and thereby ensure their better work performance. It can be assumed that if employees are more engaged, their work performance will be higher. The main goal of the paper is to examine whether corporate culture has an impact on the work performance of employees through the mediators of leadership and work engagement. The data necessary for the goal set in this way were obtained through a questionnaire survey, which was attended by 489 respondents during the year 2022. After the data reliability analysis, by using serial mediation with two mediators, the influence of corporate culture on work performance was verified independently, but also through the mediators of leadership and work engagement. Based on the results, it can be claimed that individual factors have a significant impact on work performance, while the influence of corporate culture mediated by leadership and work engagement was also confirmed. The influence of corporate culture, mediated only by leadership, and the independent influence of corporate culture through work engagement on work performance were also confirmed.

## 1. Introduction

In 2020, 78 million people were infected with the coronavirus disease (COVID-19), and 1.7 million people worldwide eventually succumbed to the disease (Wakaizumi et al., 2021). For the first time in recent history, we had to face a global problem like the COVID-19 pandemic. It is proven that the pandemic had an unprecedented global impact on all spheres of life of ordinary people. The health crisis had an impact, not only on the world economy, businesses, but also on people’s health and psyche (Fellman et al., 2020; Svabova et al., 2021). Ultimately, the pandemic resulted in limited population mobility (Svabova et al., 2020).

Most businesses have shortened their operating hours, temporarily closed, or started using remote work systems for working from home, online meetings, and web conferencing (Wakaizumi et al., 2021). During uncertain situations, such as during the COVID-19 pandemic, maintaining a satisfactory level of employee performance is an important area of concern for many organizations (Saleem et al., 2021).

During the 2020 pandemic years to the present, much research has focused on job performance. Among the frequent factors related to work performance during the pandemic were, e.g., fear of COVID-19 (Sarwar et al., 2022), remote work (Adekoya et al., 2022; Kirchner et al., 2021; Toscano and Zappalà, 2021; Kifor et al., 2022), sleep patterns (Zhao et al., 2022), communication (Amano et al., 2021), burnout syndrome (Raja et al., 2022), or stress (Kumar et al., 2021), etc.

However, our research focuses on organizational factors such as corporate culture (Tulcanaza-Prieto et al., 2021; Alkhadra et al., 2022), leadership (Nauman et al., 2020; Pancasila et al., 2020; Zheng et al., 2020), and work engagement (Eguchi et al., 2020; Neuber et al., 2022; Supanto et al., 2022). The areas of corporate culture, leadership, work engagement, and work performance can be considered sufficiently researched. However, it is interesting to examine the relationships between the mentioned factors. Chen et al. (2015), Nauman et al. (2020), Zheng et al. (2020), and Alkhadra et al. (2022) investigated the influence of leadership, or a specific type of leadership, on the work performance of the company or the work performance of employees. Since the performance of the company reflects the performance of its employees, the results of these studies can be considered as relevant for our research. Supanto et al. (2022) investigated the influence of leadership on work efficiency. If employees are efficient, their work performance increases, and, as a result, the performance of the organization also increases. Mathew et al. (2012), Guiso et al. (2015), and Tulcanaza-Prieto et al. (2021) in turn, investigated the relationship between corporate culture and company performance. Its subsequent value also depends on the performance of the company, and for that reason, the influence of corporate culture on the value of films was addressed by Huang et al. (2015). The effect of work engagement on work performance was examined more closely by Eguchi et al. (2020) and Neuber et al. (2022). Several factors were dealt with by Pancasila et al. (2020), who investigated the impact of motivation and leadership on job satisfaction and the subsequent impact on work performance. In Table 1, the research objects, results, and used methods of the authors, which we followed up on, are presented.

**Table 1:** Research objectives, results and used methods of the authors reviewed.

Authors	Object of research	Results	Methods
Abdullah et al. (2021)	Explore how counterproductive work behaviour affects firm performance. At the same time, the mediating role of organizational culture and its influence on CWB and firm performance is considered.	CWB and organizational culture significantly influence firm performance both directly and indirectly. In addition, organizational culture partially mediates the relationship between CWB and corporate performance.	Pearson moment correlation; Structural equation modelling
Alkhadra et al. (2022)	Investigation of the effect of ethical leadership on organizational performance, with the mediating role of corporate social responsibility (CSR) and organizational culture.	Organizational culture and CSR are also favourably impacted by ethical leadership, in addition to organizational performance. Additionally, the connection between moral leadership and corporate performance is highly mediated by CSR and organizational culture.	Analysis of a moment structure
Doukas and Zhang (2021)	Exploration the role of corporate social culture in mergers and acquisitions and how its interaction with managerial ability affects the outcomes of M&A decisions carried out by managers of heterogeneous abilities.	Companies with talented managers are more likely to develop a corporate social culture than those with low-ability managers. Furthermore, we show that acquiring firms with stronger social culture commitment and high-ability managers typically generate atypical announcement period returns that are significantly positive and have higher post-merger performance.	Cross-sectional regression analysis; Univariate analysis
Eguchi et al. (2020)	Investigation the association between work engagement and work performance.	Increased productivity may result from increased work engagement. Women may be more affected by job engagement than men are, in terms of how well they perform at work.	Multiple regression analysis
Guiso et al. (2015)	Which dimensions of corporate culture are related to a firm's performance and why.	When employees perceive top managers as trustworthy and ethical, a firm's performance is stronger.	Regression analyses
Huang et al. (2015)	Investigation of the role of corporate culture in family firms and its implications for firm value.	Family firms exhibit a human-capital-enhancing culture that improves firm performance.	Two-stage Least Squares (2SLS) regressions and dynamic GMM regressions
Chen et al. (2015)	Exploration the relationship among emotional intelligence, perceived transformational leadership and work performance.	Work performance and emotional intelligence were positively correlated, and perceived leader's transformational leadership was found to be a positive moderator of this association.	Confirmatory factor analysis
Mathew et al. (2012)	Identifying and analysing the ways in which organizational culture and particular work outcomes impact on organizational performance.	According to the proposed framework, corporate culture has an impact on employee happiness, productivity, and quality of work.	SEM
Nauman et al. (2020)	Examine if dictatorial supervision could affect employees' performance on the workplace by causing them to withdraw from their jobs. Additionally, we look into whether the effectiveness of work-life balance might mitigate the negative impacts of authoritarian supervision on work withdrawal.	The mediating influence of job withdrawal behaviour, which is increased under authoritarian leadership, on workers' performance. Employees with higher levels of work-life balance showed a reduced influence of autocratic leadership on job performance as measured by work withdrawal behaviour.	Regression analysis
Neuber et al. (2022)	Investigation of work engagement effected the performance of employees' tasks.	Across all subcomponents of engagement, work engagement has a considerable beneficial impact on employees' task performance in all geographic locations.	Correlation analyses; Sensitivity analyses; Moderator analyses
Pancasila et al. (2020)	Determination of the effect of work motivation and leadership on job satisfaction and its implications on employee performance.	Leadership and work motivation have a positive and significant effect on job satisfaction.	Structural equation modelling
Supanto et al. (2022)	Analyse the effects of the principal's democratic leadership style, the calibre of the teachers, workplace rules, and other factors on the effectiveness of the teachers during the pandemic.	Teacher performance is significantly impacted by the principal's democratic leadership style, the quality of the teachers, work environment, and workplace discipline.	Multiple linear regression analysis
Tulcanaza-Prieto et al. (2021)	Impact of organizational culture on corporate performance.	Statistically positive relationship between organizational culture and firm performance.	Regression analysis
Zheng et al. (2020)	Examine employee work engagement as a key moderator of connection and investigate the role that task-based management and professional abilities play in influencing the indirect link between service leadership and service performance through work engagement.	The task-based professional abilities of the leader play a moderating influence, whereas management skills do not. Particularly, when leaders exhibit high levels of task-based professional abilities, the indirect influence of service leadership on service performance via job engagement is larger.	Intraclass correlation coefficients; Confidence intervals; Monte Carlo method

Our study linked to the research of the mentioned authors, and from their works, it was derived the model that is examined in our study. It is an examination of whether leadership and work engagement mediate the influence of corporate culture on the work performance of employees. Due to the fact, that in the studies reviewed, there is no information about the links created between the investigated variables, it is possible to consider this research as unique. To the best of our knowledge, no prior studies have examined the mediating role of leadership and work engagement in the relationship of corporate culture on work performance. Thus, the main goal of the paper is to examine whether corporate culture has an impact on the work performance of employees through the mediators of leadership and work engagement.

## 2. Literature review

### 2.1. Corporate culture

Culture as an informal institution has a wide and deep influence on people’s thinking, behavior and economic activities. Corporate culture is considered a prevalent system of social control (Wan et al., 2020). Social environment and its interactions with individuals can influence the quality of an individual’s motivation, subsequent behavior, and psychological wellbeing (Jo et al., 2020).

Organizational culture is considered as an elementary element of knowledge management. Employees are considered as the most important asset of the company because they directly contact customers and competitors (Tulcanaza-Prieto et al., 2021). Doukas and Zhang (2021) considered corporate culture as an important intangible asset of a company. It also represents a competitive advantage for the company. In addition, investments in building corporate culture are a manifestation of the company’s goal to be a good corporate citizen. Tulcanaza-Prieto et al. (2021) defined organizational culture as a set of differentiated elements between organizations, including customs, norms, rules, symbols, ideologies, beliefs, rituals and myths.

According to Nwibere (2013) research, democratic leadership style is significantly and favorably influenced by corporate cultures that are competitive, entrepreneurial, and consensual. For instance, they discovered that the Laissez-Faire leadership style is significantly and favorably influenced by entrepreneurial and consensual corporate cultures. Autocratic leadership style was found to be significantly and favorably influenced by bureaucratic and consensual organizational cultures. Thus, we propose:

*H1*: Corporate culture has an impact on company leadership.

Thus according research by Brenyah and Darko (2017), accomplishment and support cultures have a considerable positive impact on employee engagement in the Ghanaian public sector, while power cultures have a major negative impact. A larger percentage of critical personnel may be retained and engaged by organizations that create cultures that are in line with their members’ beliefs, according to research by Allen (2010) and his Person-Environment (P-E) Fit Model. Therefore, we state:

*H2*: Corporate culture has an impact on the work engagement of employees.

Based on the findings of Purwanto (2020) study, it is possible to draw the conclusion that business culture and transformational leadership have a good and substantial influence on job performance directly and using creative labor practices as a form of mediation. It signifies that the company’s creative work behavior and work performance will be greater the more favorable the boss’s leadership techniques and culture are. Hidayat (2017) concluded that there is a positive effect on employee performance among corporate culture variables. According to Uddin et al. (2013), there is a significant link between corporate culture and financial success. In terms of internal performance (innovation capability and interpersonal relationships), Polychroniou and Trivellas (2018) discovered a favorable correlation between cultural strength and business results (profitability, growth and reputational assets). Culture imbalance, on the other hand, has a detrimental effect on how well a company performs. According to Jin et al. (2019), an organization’s sustainability orientation is facilitated by its innovation culture, and the opposite is also true. Research such as Praditya (2020), Zuraik and Kelly (2019), or Naguib and Naem (2018) supports this conclusion, Sivakami and Samitha (2018), Syafii et al. (2015). Thus, we propose:

*H4*: Corporate culture has an impact on the work performance of employees.

Lorincová et al. (2022) considered corporate culture as the company’s personality. It reflects human dispositions, thinking, and behavior of people in the company. The results of the study by Abdullah et al. (2021) demonstrated that corporate work behavior and organizational culture significantly influence firm performance both directly and indirectly. Furthermore, organizational culture partially mediates the relationship between corporate work behavior and performance. Thus, we propose:

*H7*: Leadership mediates the impact of corporate culture on employee performance.

According to Luo et al. (2017) findings, the corporate culture significantly improves both job performance and the psychological capital characteristics of self-efficacy, optimism, hope, and resilience. The intermediate influence between corporate culture and labor performance includes psychological capital. The findings of this study have some ramifications for management strategies that use company culture to enhance performance and build psychological capital among staff members. Therefore, we state:

*H8*: Work engagement mediates the impact of corporate culture on employee performance.

According to hypothesis testing, Leadership, Organizational Culture, Work Motivation, and Job Satisfaction all have a direct impact on Performance. However, the factors of leadership and organizational culture do not directly affect the organizational mechanism (Arif et al., 2019). Thus, we propose:

*H9*: Leadership and work engagement mediate the influence of corporate culture on employee work performance.

### 2.2. Leadership

The outbreak of the COVID-19 pandemic has highlighted the importance of leadership style in achieving business performance. If employees are productive and perform quality work, the company’s performance gradually increases (Meiryani et al., 2022). Thus, the right leadership style can indicate the success of the agenda and explain quality performance (Alkhadra et al., 2022). Thus, we propose:

*H3*: Leadership has an impact on the work engagement of employees.

The main difference between a leader and a manager is in the role of the leader, i.e., proactively engaging with the best interests of individual team members to ensure the facilitating of team members development to their full potential. Traditional management is more interested in the market and financial potential of the enterprise as a whole, management is more focused on employees and is characterized by an oriented approach that has quite different connotations (Faulks et al., 2021).

The behavior of leaders shapes the behavior of organization members. Leaders are seen as a representative example of the company and at the same time have the authority to evaluate the performance of members or make decisions regarding their promotion (Lai et al., 2020). For this reason, leaders should behave ethically and serve as role models for their employees. Subsequently, employees will imitate their good behavior and standards (Zhuang et al., 2022). This is just one aspect of how leaders can influence employees. In addition, a natural leader must build respect, trust, an appropriate level of communication with employees, and must promote corporate culture and shared values (Slavković et al., 2021).

The findings indicate that while organizational culture, work environment, and leadership style all have positive and substantial effects on job satisfaction, only the leadership style has a positive and significant impact on employee performance (Pawirosumarto et al., 2017). Arif et al. (2019) Performance has been found to be directly impacted by leadership. The effectiveness of the school principal is directly related to the effectiveness of the faculty, support personnel, and students. As a result, the leadership style of the school’s principal has a significant impact on how well every employee performs. Therefore, we state:

*H5*: Leadership has an impact on the work performance of employees.

Leaders are very integral in providing important resources that help employees engage at work and further inspire work engagement through role modeling processes (Zheng et al., 2020). An important task of leaders is therefore the motivation of employees. By trying to motivate subordinates to focus on the vision and mission of the organization, to prioritize group interests, they achieve that they go beyond personal interests (Meiryani et al., 2022). Leadership is influenced and determined by the organizational culture and the experience (Hurduzeu, 2015).

Imran et al. (2020), Kaya and Karatepe (2020), or Chen et al. (2015) confirmed that there is a significant relationship between leadership and job performance. Humborstad et al. (2014) demonstrated that if leaders try to consolidate their position or strengthen it, it can have a negative impact on employee performance. Kirchner et al. (2021) confirmed that not only the work of employees during COVID-19 was difficult, but also the work of managers. Many of the managers had no experience working remotely.

### 2.3. Work engagement

Understanding the basic process of motivation is important, as motivation is considered as a critical component shaping the behavior of company employees. For this reason, it is essential for managers to understand the elementary motivating processes of members to fulfill well the requirements of the given job positions they hold (Lai et al., 2020). In the case of extrinsic motivation, satisfaction does not come because of the activity itself. It is a consequence of external factors leading to activity. A typical example of extrinsic motivation is a financial reward acting as a significant motivational factor primarily in manual work. However, it is not a general rule. When creating a motivational system, the individual characteristics of the employee profile must therefore be taken into account (Majerova et al., 2021). van Tuin et al. (2020) demonstrated that motivation positively affects work engagement. According to the JD-R model, work engagement arises through a motivational pathway, while available work resources help employees cope with the demands of their work and continue to engage in their work (Zheng et al., 2020). Neuber et al. (2022) say that highly engaged employees have positive work results and performances. Inam et al. (2021) demonstrated that work engagement can enter in relation to work performance as a mediator between work performance and self-confidence. Thus, we propose:

*H6*: Work engagement has an impact on the work performance of employees.

When engaged, the worker is employed and expresses himself physically, mentally, emotionally, or cognitively while fulfilling the role (Slavković et al., 2021). Engaged employees demonstrate active involvement in work tasks or roles. Work roles are then performed with a high level of cognitive and emotional relatedness (Slavković et al., 2021). Engagement captures, among other things, whether employees perceive their work as stimulating, meaningful, and engaging and whether they want to invest their time and energy in work (Zheng et al., 2020). Disengaged employees mainly show withdrawal and defensiveness during the performance of the role. Engaged employees show a high level of attention, connectedness, integration, or focus on performing their tasks (Lai et al., 2020).

### 2.4. Work performance

Meriyani et al. (2022) characterized employee performance as results and achievements at work. Performance is related to following a plan, with the individual focusing on a specific outcome. Work performance, which refers to how well a job is done, is influenced by general factors such as the work environment and individual factors such as the physical demands of tasks, stress levels, and extended working hours (Wakaizumi et al., 2021). Businesses cannot maintain consistency in their performance if the external conditions are unstable. The instability of the external environment causes stress, which leads to a decrease in work performance (Saleem et al., 2021). Employee performance is one of the aspects that can influence the success of the company (Meiryani et al., 2022). According to Sengottuvel and Aktharsha (2016), all aspects of organizational culture account for a sizable portion of the diversity in performance.

COVID-19 caused a deterioration in the work performance of employees. On the other hand, however, it caused an improvement in adaptive skills, as employees were forced to quickly adapt to new, rapidly emerging situations (Saleem et al., 2021). During the COVID-19 pandemic, the work performance of remote workers (employees working from home) depended mainly on individual personality, but also on the organizational context, including factors such as corporate culture, technical support, manager trust, human resources support, financial support for working from home, and training for working from home (Slavković et al., 2021).

Meriyani et al. (2022), in turn, demonstrated that during the COVID-19 pandemic, the performance of employees was not affected by leadership style, but remote work had a significant impact. It is necessary to realize that individual members of the work group work flexibly, which is why remote work leads to irregular work patterns. Each person has different goals and priorities, such as taking care of children or taking care of relatives. Working from home can develop empathy among individual team members, as it allows everyone to understand each other’s situation and avoid potential hatred (Phillips, 2020). This area deserves more detailed research, as working from home has become an integral part and an invaluable benefit after the global pandemic of COVID-19.

## 3. Methodology

The data for the research were obtained through the method of inquiry. Specifically, an online questionnaire was used to collect data for this survey. The online survey consisted of 31 questions. The questionnaire consisted of a general part, where the respondents indicated their gender, age, highest level of education, and SK NACE. Subsequently, questions on work atmosphere, corporate culture, work engagement, leadership, communication, information, motivation, conflicts, and bullying at the workplace were conceptualized. The questionnaire was filled out by 489 respondents, employees of companies operating in the territory of the Slovak Republic from various sectors of the economy. Data were collected from August 1, 2022, to October 1, 2022. IBM SPSS Statistics 25 was used to analyze all the data collected through the online questionnaire.

In terms of gender, 50.92% of the respondents were women, which represented a total of 249 respondents, and 49.08% were men, which is 240 people. The most numerous group were persons aged 26–45, with a total of 234, which represents up to 47.85% of the total number of respondents. People aged 18–25 had the third highest representation with a number of 102 (20.86%). Employees aged 46 and over will look at our research with 31.29% participation, which means 153 people. Most women came from the 26–45 age group (115), as did most men (119). Most people worked in microenterprises (167), up to 34.15%. This is not a surprising finding, as enterprises falling into smaller size categories predominate on the territory of the Slovak Republic. One hundred and twenty-eight respondents (2618%) worked in large enterprises, 106 respondents (21.68%) worked in small enterprises, and only 88 respondents worked in medium-sized enterprises, which represented 18%. Men and women mostly worked in microenterprises, while people aged 18–25 were mostly employed in medium-sized enterprises, and respondents aged 26 and over worked mostly in microenterprises. In terms of economic sectors, most respondents worked in the fields of electricity, gas, steam, and cold air supply (56 respondents; 11.45%). The construction sector (49; 10.02%), transport and storage (47; 9.61%), and industrial production (44; 9%) also had high representation. The fewest respondents worked in the fields of art, entertainment, and recreation—only 23 respondents (4.70%) and in the education sector (28; 5.73%).

The internal consistency of answers and individual questions was checked using Cronbach’s alpha, which is the most frequently used method for determining the reliability of data in the social sciences as well (Drost, 2011; Dunn et al., 2014; Cho, 2016; Bardhoshi and Erford, 2017; McNeish, 2018). In our study, the level of Cronbach’s alpha was determined at the level of 0.8, which can be considered as sufficient (good) internal consistency (Kalkbrenner, 2021).

In research over time, due to the evolution of knowledge, it is not enough to test causal hypotheses. It is necessary to deal with what spans the causal relationship. By examining whether variable X can predict or cause variable Y, researchers have increasingly used the mediation process in social psychological research (Pieters, 2017; Memon et al., 2018; Rasoolimanesh et al., 2021). Mediation can be understood as a process or a mechanism that helps us explain or describe the investigated relationship between the dependent variable Y and the independent variable X through another variable M, which is referred to as the mediator. The mediator explains the relationship between variable X and Y (Abu-Bader and Jones, 2021). In our research, a serial mediation with two mediators was implemented using the IBM SPSS Statistics 25 program. The description of the individual variables entering the serial mediation is shown in Table 2.

**Table 2:** Variable type.

Variable name	Variable type	Note
Corporate culture	Independent variable	X
Work performance	Dependent variable	Y
Leadership	Mediator variable	M1
Work engagement	Mediator variable	M2

**Figure 1:**
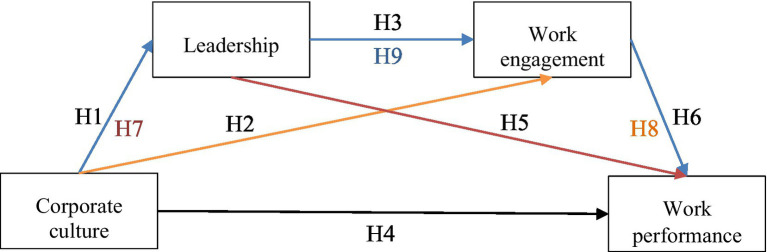
Diagram of serial mediation with two mediators according to the research variables and hypothesis.

According to Preacher and Hayes (2008), a mediation model describes how, or by what means, an independent variable (X) affects a dependent variable (Y) through one or more potential intervening variables or mediators (M). Figure 1 represents mediation model with two mediators according to hypotheses set above.

Using the model, hypotheses were established that will examine the impact of corporate culture on work performance independently, but also through two mediators (leadership and work engagement). The entire model is performed at the significance level α = 0.05.

## 4. Results

The reliability analysis in Table 3 showed, based on the value of Cronbach’s Alpha, that the data of the analyzed variables can be considered reliable with sufficient internal consistency. Four variables were processed, namely corporate culture, leadership work commitment, and work performance.

**Table 3:** Results of reliability analysis.

Reliability statistics	
Cronbach’s alpha	N of items
0.805	4

A serial mediation model was used in IBM SPSS Statistics 25 using Hayes’ PROCESS macro for SPSS (Hayes, 2022). The data in Table 4 show the influence of corporate culture on leadership, while the value of the coefficient is 0.663. The value of p of the test is zero and is less than the significance level α = 0.05. From this it can be concluded that hypothesis H1 is confirmed. Both the lower and upper confidence intervals are greater than 0, and therefore, the effect is verified to be significant.

**Table 4:** Influence of corporate culture on leadership.

Outcome variable: Leadership						
Model summary						
*R*	*R* ^2^	MSE	*F*	df1	df2	*p*
0.592	0.351	0.801	262.914	1.000	487.000	0.000
Model						
	Coeff.	SE	*t*	*p*	LLCI	ULCI
Constant	1.186	0.168	7.046	0.000	0.856	1.517
Corporate Culture	0.663	0.041	16.2145	0.000	0.582	0.743

Table 5 shows the impact of the corporate culture variable along with leadership on the company’s work engagement. In the first case (the influence of corporate culture on work engagement), the coefficient value is 0.116 and the standard error value is 0.050. The value of p of the test is zero, and therefore the hypothesis H2 can be accepted. Since the values of the upper and lower confidence interval are positive, it is a significant relationship. The relationship between leadership and work engagement is also considered significant at a significance level of 0.05, as the value of the confidence intervals is greater than 0. At the same time, hypothesis H3 is confirmed, as the value of p is 0.021, which is less than 0.05. The coefficient has a value of 0.297 and the standard error is 0.045.

**Table 5:** Influence of corporate culture and leadership on work engagement.

Outcome variable: Work engagement						
Model summary						
R	*R* ^2^	MSE	*F*	df1	df2	*p*
0.422	0.178	0.775	52.512	2.0000	486.0000	0.0000
Model						
	Coeff.	SE	*t*	*p*	LLCI	ULCI
Constant	2.377	0.174	13.678	0.000	2.035	2.718
Corporate culture	0.116	0.050	2.319	0.021	0.018	0.214
Leadership	0.297	0.045	6.671	0.000	0,210	0.385

The values in Table 6 show that, based on the value of p of the variables corporate culture, leadership, and work engagement, it is possible to claim that the mentioned variables have an impact on work performance. The *p*-value of all three variables is zero and since it is less than the significance level α = 0.05, we can accept hypotheses H4, H5 and H6. At the same time, it is obvious that these are significant dependencies. The value of the coefficient for corporate culture is 0.476 with a standard error of 0.142. The coefficient of leadership reaches a value of 0.224 with a standard error of 0.032. The last variable work engagement has a value of 0.134 with a standard error of 0.032.

**Table 6:** Influence of corporate culture, leadership, and work engagement on work performance.

Outcome variable: Work performance						
Model summary						
*R*	*R* ^2^	MSE	*F*	df1	df2	*p*
0.759	0.577	0.374	220.251	3.000	485.000	0.000
Model						
	Coeff.	SE	*t*	*p*	LLCI	ULCI
Constant	0.814	0.142	5.724	0.000	0.534	1.093
Corporate culture	0.476	0.035	13.670	0.000	0.408	0.545
Leadership	0.224	0.032	6.925	0.000	0.160	0.288
Work engagement	0.134	0.032	4.255	0.000	0.072	0.196

Based on the previous results, we have confirmed 6 established hypotheses so far. Table 7 contains data on the last three hypotheses. The overall effect of the given model is significant based on LLCI and ULCI values. The direct effect of variable X, in our case represented corporate culture, has a significant impact on the resulting variable Y (employee performance). To confirm the hypotheses H7; H8 and H9 it is necessary to focus on the indirect effect of variable X on variable Y. The total value of the indirect effect is 0.190. If added to the direct effect, it represents the total effect. The value of the indirect effect consists of three variables. The variable Ind1 represents the relationship between corporate culture, leadership, and work performance. Since both the upper and lower confidence intervals contain only positive numbers, this is a significant effect and the validity of hypothesis H7 can be confirmed. Ind2 represents the relationship between corporate culture, work engagement, and employee performance. The limits of the upper and lower interval are positive, so we accept the hypothesis H8 as well. Ind3 represents the relationship between variable X, M1, M2 and Y, and thus between corporate culture, leadership, work engagement, and work performance. Even in the last case, the values of the confidence interval are positive, and therefore, the hypothesis H9 is accepted. According to the individual value of the effects, the highest effect has the indirect effect of Ind1. So, it is about the relationship between corporate culture, leadership, and work performance.

**Table 7:** Total effect of company culture on work performance.

Total effect of X on Y					
Effect	SE	*t*	*p*	LLCI	ULCI
0.667	0.030	21.958	0.000	0.607	0.726
**Direct effect of X on Y**					
Effect	SE	*t*	*p*	LLCI	ULCI
0.476	0.035	13.670	0.000	0.408	0.545
**Indirect effect (s) of X on Y**					
	Effect	Boot SE	Boot LLCI	Boot ULCI	
TOTAL	0.190	0.028	0.138	0.247	
Ind1	0.148	0.027	0.097	0.204	
Ind2	0.016	0.010	0.001	0.037	
Ind3	0.026	0.009	0.010	0.044	

**Figure 2:**
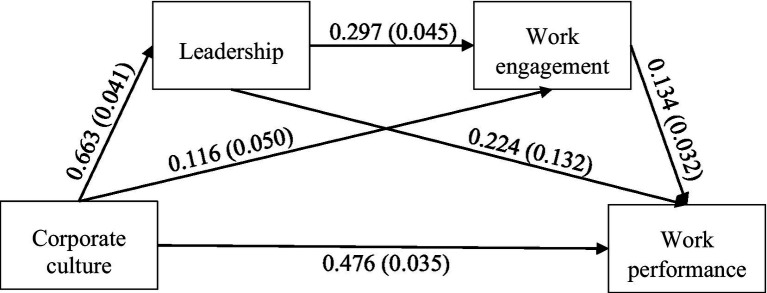
Path diagram of serial mediation with two mediators.

Based on the above results, a diagram (Figure 2) is constructed for serial mediation with two mediators, but this time with added values of coefficients and standard errors.

## 5. Discussion

Several studies partially match our results. In a sample of 300 respondents working in biomedical companies, Abdullah et al. (2021) demonstrated that counterproductive work behavior and organizational culture significantly affect firm performance both directly and indirectly. We agree with the authors’ conclusions, as we have also demonstrated the impact of corporate culture on work performance, either directly or indirectly through leadership, work engagement, or both at the same time. Alkhadra et al. (2022) focused on determining the impact of a specific type of leadership, namely ethical leadership, on corporate culture and CSR. Their assumption was confirmed, while, for example, they found that CSR and corporate culture mediate the relationship between ethical leadership and organizational performance. Tulcanaza-Prieto et al. (2021) in their research demonstrated a statistically significant positive relationship between corporate culture and business performance. Since the performance of the organization is the result of the performance of the employees, we can claim that we achieved the same results mentioned by the research of both authors. The only difference is that we have demonstrated the direct influence of leadership on the organization and, at the same time, the mediation of the influence of corporate culture on work performance. Alkhadra et al. (2022), in turn, demonstrated that the influence of ethical leadership is mediated by corporate social responsibility.

Doukas and Zhang (2021) looked at the issue from the opposite point of view. They provided strong evidence that a high level of leadership tends to build a corporate social culture. In addition, they documented the fact that if companies had a higher engagement in the area of social culture under the leadership of a strong and capable leader, then the companies achieve high returns, which ultimately reflects that the work performance of the employees is high. Doukas and Zhang (2021) demonstrated the influence of leadership on corporate culture, in contrast to our research, where we observed the relationship of the mentioned variables from the opposite point of view. This is an interesting insight, as it is possible that the mentioned two factors influence each other. Huang et al. (2015) demonstrated that a specific form of corporate culture, namely the culture of strengthening human capital, improves performance. A strong link between corporate culture and work performance was also demonstrated by Mathew et al. (2012). We agree with the results of the authors, as we have demonstrated in the research by whether direct or indirect (through leadership, work engagement, or both at the same time) influence of corporate culture on work performance.

Chen et al. (2015) demonstrated that leadership acts as a mediator, for example, between emotional intelligence and job performance. It follows that leadership has an impact on work performance. Also, as in our research, also in the research of Chen et al. (2015), however, it is an intermediary relationship. In both studies, leadership mediates the effect on work performance for another factor. Guiso et al. (2015) confirmed in their studies that performance increases if employees perceive top management as trustworthy and ethical. Based on the results, it can be concluded that as long as there is a culture in the company that builds credibility and is based on morals, while it is also supported by the managers themselves in the form of leadership, then the performance of the employees grows. As part of the research, we came to the same conclusions.

Prochazka et al. (2017) demonstrated that there is a connection between transformational leadership and group work performance, which is mediated by job satisfaction. The authors’ results can be considered as support to our results. However, the fundamental difference lies in the factor that enters the model as a mediator. The authors appropriately used job satisfaction, which can be considered a factor affecting not only work performance, but also work engagement. According to Pancasila et al. (2020), leadership has a more significant direct impact on employee performance than the indirect impact of leadership on employee performance through job satisfaction. Zheng et al. (2020) pointed out that when executives exhibit a high level of task-based professional skills related to leadership, the indirect effect of service leadership on service performance is greater. The authors pointed to the fact that if management has a high level of professional skills, the indirect influence of leadership is more pronounced. We cannot substantiate this fact, since we did not examine the influence of factors other than corporate culture on leadership. Toward the future, this is an interesting insight that would be appropriate to implement.

In the area of the impact of work engagement on work performance, Eguchi et al. (2020) presented the same results as we did in our study. Higher work engagement can therefore have a positive effect on work performance, and at the same time, it has been shown that the impact of work engagement on work performance can be greater for women than for men. It can be considered as an advantage of the study that the investigated issue was also addressed from the point of view of gender. In our case, we did not devote ourselves to a more detailed investigation, either from the point of view of gender, age, or the size of the company, as in our opinion, a larger sample would be needed for this type of research. Work engagement is positively associated with future task performance and negatively with future absenteeism (Neuber et al., 2022).

## 6. Conclusion

Corporate culture as the cornerstone of shaping employee behavior can currently be considered as a competitive advantage of the company. However, corporate culture not only shapes the behavior of employees, but also creates working conditions that have an impact on work performance. Managers themselves are also responsible for the behavior and motivation of employees. Not only do they manage employees, but they have to bring individual goals closer to them and motivate them so that the goals are fulfilled. To be able to do this, the manager must also be a good leader, whom the employees are willing to follow. Working conditions, but also leadership, should have an impact on employee engagement itself. If employees are engaged, it can be assumed that their work performance will have an upward trend.

The main goal of our study was to investigate whether the corporate culture has an influence through the mediators of leadership and work engagement on the work performance of employees, we consider it fulfilled. We examined the reliability of the data, which was confirmed, and subsequently, we were able to verify the established hypotheses through serial mediation with two mediators. Based on the data obtained from 489 respondents, all nine hypotheses were proven. The most significant output of our study was the confirmation of the last hypothesis, as follows: Leadership and work engagement mediate the influence of corporate culture on the work performance of employees. In addition, we found that both leadership and work engagement act independently as a mediator in the relationship between corporate culture and work performance.

The fact that more than 34% of the obtained data came from micro-enterprises can be considered as a limitation of the research. These data can be more distorted, as a family atmosphere and friendly relations prevail in micro-enterprises.

The practical benefit of this study is that the findings can be used by managers, but also by the companies themselves, as a guide for improving the performance of employees. If companies will work on improving corporate culture and managers on improving their leadership skills, they can increase the work engagement of employees, which will ultimately improve the work performance of employees.

To the best of the authors’ knowledge, this research publication is the first document that focused on analyzing the impact of corporate culture on the work performance of employees through two mediators of leadership and work engagement, which can represent a cornerstone for subsequent research into mutual basic relational relationships, or a stimulus for investigating value-creating relations using moderation or mediation of the variables investigated in this study or by adding other relevant variables from the field of work performance, even in a crisis period.

## Data availability statement

The raw data supporting the conclusions of this article will be made available by the authors, without undue reservation.

## Ethics statement

Ethical review and approval was not required for the study on human participants in accordance with the local legislation and institutional requirements. Written informed consent for participation was not required for this study in accordance with the national legislation and the institutional requirements.

## Author contributions

All authors listed have made a substantial, direct, and intellectual contribution to the work and approved it for publication.

## Funding

This paper is an output of scientific project VEGA no. 1/0032/21: Marketing engineering as a progressive platform for optimizing managerial decision-making processes in the context of the current challenges of marketing management and an output of the project NFP313010BWN6 “The implementation framework and business model of the Internet of Things, Industry 4.0 and smart transport.”

## Conflict of interest

The authors declare that the research was conducted in the absence of any commercial or financial relationships that could be construed as a potential conflict of interest.

## Publisher’s note

All claims expressed in this article are solely those of the authors and do not necessarily represent those of their affiliated organizations, or those of the publisher, the editors and the reviewers. Any product that may be evaluated in this article, or claim that may be made by its manufacturer, is not guaranteed or endorsed by the publisher.
